# Imaging of CAIX-expressing xenografts *in vivo* using ^99m^Tc-HEHEHE-ZCAIX:1 Affibody molecule

**DOI:** 10.3892/ijo.2014.2782

**Published:** 2014-11-27

**Authors:** HADIS HONARVAR, JAVAD GAROUSI, ELIN GUNNERIUSSON, INGMARIE HÖIDÉN-GUTHENBERG, MOHAMED ALTAI, CHARLES WIDSTRÖM, VLADIMIR TOLMACHEV, FREDRIK Y. FREJD

**Affiliations:** 1 Unit of Biomedical Radiation Sciences, Rudbeck Laboratory, Uppsala University, SE-75185 Uppsala, Sweden; 2 Affibody AB, SE-17163 Solna, Sweden; 3 Department of Hospital Physics, Uppsala University Hospital, SE-75185 Uppsala, Sweden

**Keywords:** CAIX, affibody molecules, radionuclide imaging, technetium-99m, HEHEHE-tag, hypoxia, xenografts

## Abstract

Carbonic anhydrase IX (CAIX) is a transmembrane enzyme involved in regulation of tissue pH balance. In cancer, CAIX expression is associated with tumor hypoxia. CAIX is also overexpressed in renal cell carcinoma and is a molecular target for the therapeutic antibody cG250 (girentuximab). Radionuclide imaging of CAIX expression might be used for identification of patients who may benefit from cG250 therapy and from treatment strategies for hypoxic tumors. Affibody molecules are small (7 kDa) scaffold proteins having a high potential as probes for radionuclide molecular imaging. The aim of the present study was to evaluate feasibility of *in vivo* imaging of CAIX-expression using radiolabeled Affibody molecules. A histidine-glutamate-histidine-glutamate-histidine-glutamate (HE)_3_-tag-containing CAIX-binding Affibody molecule (HE)_3_-ZCAIX:1 was labeled with [^99m^Tc(CO)_3_]^+^. Its binding properties were evaluated *in vitro* using CAIX-expressing SK-RC-52 renal carcinoma cells. ^99m^Tc-(HE)_3_-ZCAIX:1 was evaluated in NMRI nu/nu mice bearing SK-RC-52 xenografts. The *in vivo* specificity test confirmed CAIX-mediated tumor targeting. ^99m^Tc-(HE)_3_-ZCAIX:1 cleared rapidly from blood and normal tissues except for kidneys. At optimal time-point (4 h p.i.), the tumor uptake was 9.7±0.7% ID/g, and tumor-to-blood ratio was 53±10. Experimental imaging of CAIX-expressing SK-RC-52 xenografts at 4 h p.i. provided high contrast images. The use of radioiodine label for ZCAIX:1 enabled the reduction of renal uptake, but resulted in significantly lower tumor uptake and tumor-to-blood ratio. Results of the present study suggest that radiolabeled Affibody molecules are promising probes for imaging of CAIX-expression *in vivo*.

## Introduction

Carbonic anhydrases (CA) are a family of zinc metallo-enzymes that participate in the regulation of pH, CO_2_ and HCO_3_^−^ transport as well as the water and electrolyte balance (1). The membrane associated glycoprotein carbonic anhydrase 9 (CAIX) has the highest catalytic activity among the members of the family (2) and catalyzes the reversible hydration of carbon dioxide into carbonic acid. CAIX consists of four domains, the N-terminal proteoglycan domain, the catalytic domain exposed to the extracellular milieu, a transmembrane anchor, and a short cytoplasmic tail (3). Expression of CAIX is elicited under hypoxic conditions by hypoxia inducible factor-1 (HIF-1α) (4). Overexpression of CAIX is associated with tumor cell hypoxia in a variety of human tumors (5), including breast (6), bladder (7), head and neck carcinomas (8), esophageal and gastric adenocarcinomas (9) and carcinomas of the lung (10).

A number of clinical and preclinical studies (11) have demonstrated a correlation between CAIX expression in tumors and resistance to chemotherapy and radiotherapy as well as increased potential for metastasis and poor cancer prognosis (12,13). Thus, identification of hypoxic regions within the tumors will aid the stratification of patients that may benefit from alternative treatment approaches for hypoxic tumors, such as use of radiosensitizers (14), hyperthermia (15), or hypoxia-selective cytotoxins (16). This identification requires availability of hypoxia detection assays.

Measurement of tumor hypoxia is feasible using both traditional oxygenation measurements that are based on oxygen-sensitive electrodes, and by imaging using positron emission tomography (PET), and single photon emission computed tomography (SPECT). The *in vivo* oxygenation measurement methodologies are clinically less attractive due to their invasiveness and accessibility limitations (17). Therefore, development of non-invasive approaches for imaging of regional tumor tissue hypoxia remains to be of interest. The limited normal tissue expression of CAIX (epithelia of the stomach, small intestine and gall bladder) (5) makes it an attractive target for molecular imaging, which would allow both identification of hypoxic tumors and predicting treatment outcome.

Currently, radiolabeled nitro-imidazole compounds have found a clinical application for imaging of hypoxia (18). In hypoxic cells, nitro-imidazole compounds are reduced by intracellular reductases into highly reactive intermediates, which subsequently bind to thiol groups of intracellular proteins, resulting in accumulation inside hypoxic cells (19). Multiple studies have been performed to improve *in vivo* stability of substrates with nitro-groups against enzymatic cleavage for visualization of tumor hypoxia using both SPECT (20) and PET (21). Among these hypoxia imaging agents are the fluoromisonidazole (^18^F-FMISO) (22) and the recently designed ^18^FHX4 with improved pharmacokinetic and clearance properties (23). A major challenge in development of nitro-imidazole-based imaging agents for hypoxia is the need to penetrate inside malignant cells, which requires sufficiently high lipophilicity of a tracer. A high lipophilicity slows down elimination of an unbound tracer from normal tissues, which reduces tumor to normal tissue ratio of radioactivity concentration (18). Therefore, the use of an extracellular hypoxia-associated molecular abnormality would be desirable.

Presence of the extracellular domain of CAIX makes it a potential target for specific molecular detection approaches using targeting proteins. Currently, CAIX is used clinically as a diagnostic target for antibodies with implications for both therapy and patient outcome (24). Monoclonal antibodies with high affinity, such as chimeric G250 and M75 have already been generated and tested for this purpose. Among these, M75 is useful for western blotting, immunoprecipitation, and immunohistochemistry (25) whereas the anti-CAIX antibody cG250 was mostly studied for imaging of renal clear cell carcinoma (RCC) (26). It has demonstrated also an obvious potential for *in vivo* imaging of hypoxia with high tumor specificity. A considerable effort has been made to explore its potential for immunotherapy as well (27).

Hypoxic regions are, however, distant from blood vessels (28), and an efficient targeting agent must therefore have excellent tissue penetration properties. Like any other monoclonal antibody, the cG250 has some limitations due to its large size. In addition to the relatively poor extravasation and tissue penetration, the long blood circulation of cG250 necessitates several (4–7) days interval between injection and imaging for obtaining optimal tumor uptake and high contrast images (29). Recently, a number of studies for development of targeting agents with smaller molecular weights, e.g. engineered or enzymatically produced antibody fragments (30–32) and peptides (33), have been performed to address this problem. However, there is still room for improvement.

A new class of engineered small scaffold proteins, Affibody molecules, may be an alternative tracer with favorable properties for radionuclide molecular imaging (34). Affibody molecules are composed of a 3-helix cysteine-free bundle consisting of 58 amino acids (35). Randomization of 13 amino acids in helices one and two displaces the native binding specificity and creates a large library from which high affinity binders to different proteinaceous targets are selected (35). Further affinity maturation permits development of binders with picomolar affinity to different cancer-associated molecular targets (36). The small size (7 kDa) of Affibody molecules facilitates high rates of extravasation and tissue penetration, and rapid blood clearance of unbound tracers, which provides high contrast images a few hours after injection (37). Clinical data have demonstrated high potential of Affibody molecules in molecular imaging (38,39).

The aim of the present study was to evaluate feasibility of CAIX imaging *in vivo* using radiolabeled Affibody molecules.

## Materials and methods

### General

Buffers, including 0.1 M phosphate-buffered saline (PBS), pH 7.5, and 0.07 M sodium borate, pH 9.3, were prepared using common methods from chemicals supplied by Merck (Darmstadt, Germany). High-quality Milli-Q^©^ water (resistance higher than 18 MΩ/cm) was used for preparing solutions. IsoLink kits were kindly provided by Covidien (Mansfield, MA, USA). ^99m^Tc was obtained as pertechnetate from an Ultra-TechneKow generator (Covidien) by elution with sterile 0.9% NaCl. ^125^I-sodium iodide was purchased from Perkin-Elmer (Waltham, MA, USA). Chloramine-T and sodium metabisulfite were from Sigma-Aldrich (St. Louis, MO, USA). NAP-5 size exclusion columns were purchased from GE Healthcare (Uppsala, Sweden). Cells used during *in vitro* experiments were detached using trypsin-EDTA solution (0.25% trypsin, 0.02% EDTA in buffer; Biochrom AG Biotechnologie, Berlin, Germany). For *in vivo* experiments, Ketalar (50 mg/ml; Pfizer, Inc., New York, NY, USA), Rompun (20 mg/ml; Bayer, Leverkusen, Germany) and heparin (5,000 IE/ml; Leo Pharma, Copenhagen, Denmark) were used.

Radioactivity was measured using an automated gamma-counter with a ~7.6-cm (3-in) NaI(Tl) detector (1480 Wizard; Wallac Oy, Turku, Finland). The yield and purity of radiolabeled Affibody molecules was determined by radio-instant thin layer chromatography (radio-ITLC) 150–771 Dark Green, (Tec-Control Chromatography strips from Biodex Medical Systems, Inc., Shirley, NY, USA). Sodium dodecyl sulfate polyacrylamide gel electrophoresis (SDS-PAGE), 200 V constant using NuPAGE 4–12% Bis-Tris Gel (Invitrogen AB, Lidingö, Sweden) in MES buffer (Invitrogen AB) was used for cross-validation of stability results. The distribution of radioactivity along the thin layer chromatography strips and SDS-PAGE gels was measured on a Cyclone™ Storage Phosphor system and analyzed using OptiQuant™ image analysis software (Perkin-Elmer).

Data on cellular processing and biodistribution were assessed by an unpaired, two-tailed t-test using the GraphPad Prism (version 6.00 for Windows; GraphPad Software, San Diego, CA, USA) in order to determine any significant differences (P<0.05).

Details on selection of CAIX-targeting Affibody molecule ZCAIX:1 will be reported elsewhere. A histidine-glutamate-histidine-glutamate-histidine-glutamate (HE)_3_-tag (40) was introduced at N terminus of ZCAIX:1 for site-specific labelling with ^99m^Tc.

### Labeling and stability test of Affibody molecules with [^99m^Tc (CO)_3_]^+^ and ^125^I

Radiolabelling of (HE)_3_-ZCAIX:1 with [^99m^Tc(CO)_3_]^+^ was performed as described earlier (40). Briefly, 400–500 μl (~3GBq) of ^99m^TcO_4_^−^-containing generator eluate was added to a lyophilized IsoLink kit. The mixture was incubated at 100°C for 30 min. Thereafter, 40 μl of mixture was transferred to a vial containing 100 μg of Affibody molecule in 40 μl of PBS, followed by incubation at 50°C. After incubation for 60 and 120 min, 1 μl samples were taken for analysis of the radiochemical labeling yields using radio-ITLC. When the ITLC strips were eluted with PBS, pertechnetate, as well as carbonyl and histidine complexes of ^99m^Tc, migrated with the eluent front (R_f_=1.0), while Affibody molecules did not move under these conditions (R_f_=0.0).

The radio-labeled Affibody molecules were purified using NAP-5 columns pre-equilibrated and eluted with PBS. The purity of each preparation was evaluated using radio-ITLC. Stability of the ^99m^Tc-(HE)_3_-ZCAIX:1 was tested using histidine challenge method in which paired samples of the radio-labeled conjugate were incubated at 37°C with 500-fold and 5,000-fold excess of histidine for 4 h. Control samples were treated in the same way but incubated in PBS. Thereafter, the samples were analyzed using ITLC as described above.

Indirect radio-iodination of (HE)_3_-ZCAIX:1 using N-succinimidyl-para-(trimethylstannyl)-benzoate was performed as previously described (41). Stability of the ^125^I-iodinated Affibody molecule was tested using three different challenge methods: incubation in presence of 2 M non-radioactive NaI to disrupt non-covalent adhesion of radio-iodide with the protein, in presence of 30% ethanol to disrupt hydrophobic interaction of iodobenzoic acid with the protein, and in presence of a mixture of 2 M cold NaI and 30% ethanol. Paired samples of radiolabeled conjugate were incubated at room temperature with the test solution for 4 h.

### Affinity determination using LigandTracer

The kinetics of binding of ^99m^Tc-(HE)_3_-ZCAIX:1 to living CAIX-expressing SK-RC-52 renal carcinoma cells was measured at 4°C using LigandTracer Yellow (Ridgeview Instruments, Vänge, Sweden) according to the established method (42). The LigandTracer device records in real-time kinetics of binding to and dissociation of radiolabeled tracers from living cells. TraceDrawer software (Ridgeview Instruments) permits to calculate both the association and dissociation rates and, based on that, the affinity of radiolabeled conjugates is determined. In order to cover the concentration span needed for proper affinity estimation, three increasing concentrations of ^99m^Tc-(HE)_3_-ZCAIX:1 (30, 90 and 150 nM) were used in each affinity assay.

### In vitro binding specificity and cellular processing assays

Binding specificity and cellular processing studies were performed using the renal clear cell carcinoma SK-RC-52 cell line by methodology validated earlier for anti-HER2 Affibody molecules (43).

To evaluate specificity, ^99m^Tc-(HE)_3_-ZCAIX:1 (13 nM) was added to two sets of Petri dishes containing a monolayer cell (~1×10^6^ cells/dish). To one set of the Petri dishes, a 100-fold molar excess of the unlabeled (HE)_3_-ZCAIX:1 was added ~15 min before addition of the radiolabeled conjugate to saturate binding sites. The cells were incubated in a humidified incubator (5% CO_2_, 37°C) for 1 h. Thereafter, the medium was collected, the cells were washed with cold serum-free medium and then trypsin-EDTA solution was added and incubated for 10 min. Detached cells were collected. The radioactivity of cells and media was measured and the percent of cell-bound radioactivity was calculated.

To evaluate cellular processing, SK-RC-52 cells were incubated with 13 nM ^99m^Tc-(HE)_3_-ZCAIX:1 at 37°C and 5% CO_2_. At designated time-points (1, 2, 4, 8 and 24 h), a group of three dishes was removed from the incubator, the media was collected and cells were washed with ice cold serum-free medium. Thereafter, cells were treated with 0.5 ml 0.2 M glycine buffer, pH 2.0, containing 4 M urea, for 5 min on ice. The acidic solution was collected and cells were additionally washed with 0.5 ml of glycine buffer. The acidic fractions were pooled. The cells were then incubated with 0.5 ml of 1 M NaOH at 37°C for at least 20 min. The cell debris was collected and the dishes were additionally washed with 0.5 ml of NaOH solution. The alkaline fractions were pooled. The radioactivity in the acidic solution was considered as membrane bound, and in the alkaline fractions as internalized.

### In vivo evaluation of ^99m^Tc-(HE)_3_-ZCAIX:1 and ^125^I-(HE)_3_-ZCAIX:1

Animal experiments have been performed according to national legislation on laboratory animal protection and were approved by the Ethics Committee for Animal Research of the Uppsala University (Permit Number: 48/11). Euthanasia was performed under Rompun/Ketalar anesthesia, and all efforts were made to minimize suffering.

Biodistribution studies were performed in female NMRI nu/nu mice. Two weeks before the study, 10×10^6^ SK-RC-52 cells were implanted in right hind leg of NMRI nu/nu mice. Average tumor weight was 0.30±0.14 g at the time of the experiment, and the average animal weight was 17.1±1.3 g. For biodistribution study of ^99m^Tc-(HE)_3_-ZCAIX:1, mice were randomized into groups of four. In order to determine the optimal injected protein dose, 3 groups of animals were injected intravenously (tail vein) with three different doses: 0.3 μg (80 kBq), 1 μg (80 kBq), and 5 μg (110 kBq) of ^99m^Tc-(HE)_3_-ZCAIX:1 in 100 μl PBS and sacrificed at 4 h p.i. To check the specificity of the xenograft targeting of ^99m^Tc-(HE)_3_-ZCAIX:1, a group of four mice was subcutaneously pre-injected with 500 μg non-labeled (His)_6_-ZCAIX:1 Affibody molecule 40 min before injection of 1 μg (80 kBq) of ^99m^Tc-(HE)_3_-ZCAIX:1 and the mice were sacrificed at 4 h after injection of radioactive tracer. Two additional groups of mice were injected with 1 μg (110 kBq) to measure biodistribution of ^99m^Tc-(HE)_3_-ZCAIX:1 at 1 and 8 h p.i.

To study biodistribution of ^125^I-(HE)_3_-ZCAIX:1, 6 mice were randomized into two groups of 3. Animals were injected intravenously with 1 μg (27 kBq) ^125^I-(HE)_3_-ZCAIX:1 per animal in 100 μl PBS. The biodistribution was measured at 6 and 8 h after injection.

Mice were sacrificed at predetermined time-points by an intra-peritoneal injection of anesthesia (20 μl/g body weight; Ketalar, 10 mg/ml; Rompun, 1 mg/ml). Organs and tissue samples were excised and weighed, and their radioactivity was measured. The tissue uptake values were calculated as percent of injected dose per gram tissue (% ID/g).

In vivo imaging was performed to obtain a visual confirmation of the biodistribution data. Two SK-RC-52 xenograft bearing mice were injected with 11 MBq (3 μg) of ^99m^Tc-(HE)_3_-ZCAIX:1. Mice were sacrificed by cervical dislocation at 4 h after injection. The imaging experiment was performed using an Infinia γ-camera (GE Healthcare) equipped with a low energy high-resolution (LEHR) collimator. Static images (30 min) were obtained with a zoom factor of 2 in a 256×256 matrix.

## Results

### Labeling of Affibody molecules with [^99m^Tc(CO)_3_]^+^ and ^125^I

^99m^Tc-(HE)_3_-ZCAIX:1 Affibody molecules were efficiently labeled with ^99m^Tc. The yield was 75±4%. After purification with disposable NAP-5 column, the radiochemical purity of the conjugate was 99.6±0.2%. The radioiodination yield was 14±1%, and the purity of ^125^I-(HE)_3_-ZCAIX:1 was of 98±1%.

^99m^Tc-(HE)_3_-ZCAIX:1 was stable under histidine challenge during 4 h, with no measurable release of radionuclide after incubation with both 500 and 5000-fold molar excess amounts of histidine. Similarly, no release of radioactivity from ^125^I-(HE)_3_-ZCAIX:1 was detected after incubation with NaI (2 M), 30% ethanol or a mixture of NaI (2 M) and 30% ethanol. The amount of released ^125^I was minor and within accuracy of the analytical method.

### Affinity determination using LigandTracer

The kinetic measurements of ^99m^Tc-(HE)_3_-ZCAIX:1 binding to CAIX-expressing SK-RC-52 cells *in vitro* using LigandTracer Yellow confirmed that its high affinity to CAIX was preserved after radiolabeling. The best fitting for ^99m^Tc-labeled (HE)_3_-ZCAIX:1 was obtained using a 1:2 interaction model, which indicated that the binding of this radiolabeled conjugate to living CAIX-expressing cells is mediated by two binding site populations, one with strong and one with weaker interaction. The dissociation constant at equilibrium (KD) for the first interaction was 1.3 nM, and for the second one 130 nM.

### In vitro binding specificity and cellular processing

Addition of 100-fold excess of non-labeled Affibody molecules caused a significant (P<0.005) decrease in CAIX-binding of ^99m^Tc-(HE)_3_-ZCAIX:1 to CAIX-expressing SK-RC-52 cells, from 9.6±0.1 to 0.66±0.02% of added radioactivity. This demonstrated saturable binding of radioconjugates, indicating their specific interaction.

**Figure 1:**
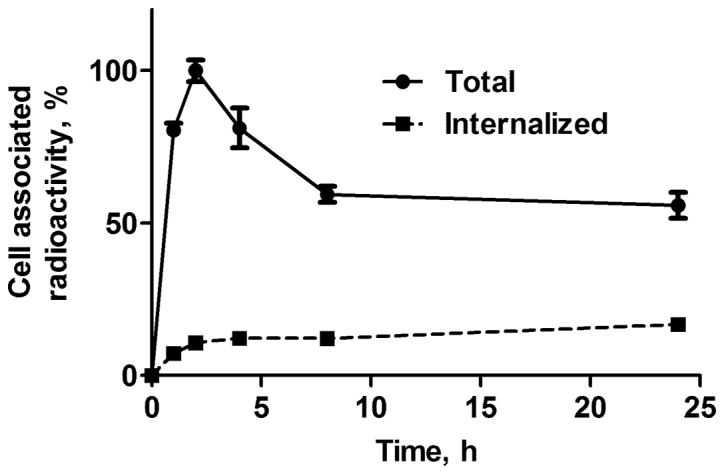
Cellular processing of ^99m^Tc-(HE)_3_-ZCAIX:1 by CAIX-expressing SK-RC-52 cells *in vitro*. Cell bound activity is normalized to the maximum uptake. Data are presented as mean values for six cell dishes and standard deviations. Error bars might be smaller than the symbols.

Cellular processing of ^99m^Tc-(HE)_3_-ZCAIX:1 by CAIX-expressing SK-RC-52 cells is presented in Fig. 1. The binding pattern of ^99m^Tc-(HE)_3_-ZCAIX:1 showed a rapid increase in total cell-associated radioactivity up to 2 h but decreased considerably at 8 h (59±2% of maximum) and remained steady up to 24 h (55±4% of maximum). To exclude experimental artifacts, this experiment was repeated twice, but demonstrated very concordant results. The internalization of all radio-conjugates was slow and increased slightly throughout the assay. The percentage of internalized radioactivity by SK-RC-52 cells at 24 h after the start of incubation was 16.7±0.1%.

### In vivo studies of ^99m^Tc-(HE)_3_-ZCAIX:1 and ^125^I-(HE)_3_-ZCAIX:1

The data concerning biodistribution of ^99m^Tc-(HE)_3_-ZCAIX:1 female NMRI nu/nu mice bearing SK-RC-52 xenografts at 4 h after injection of different protein doses are presented in Tables I and II. There was no significant difference in tumor-to-organ ratios, except that tumor-to-spleen ration was significantly lower at the injected protein dose of 0.3 μg than 5 μg. An injected dose of 1 μg was selected for further animal studies.

**Figure 2:**
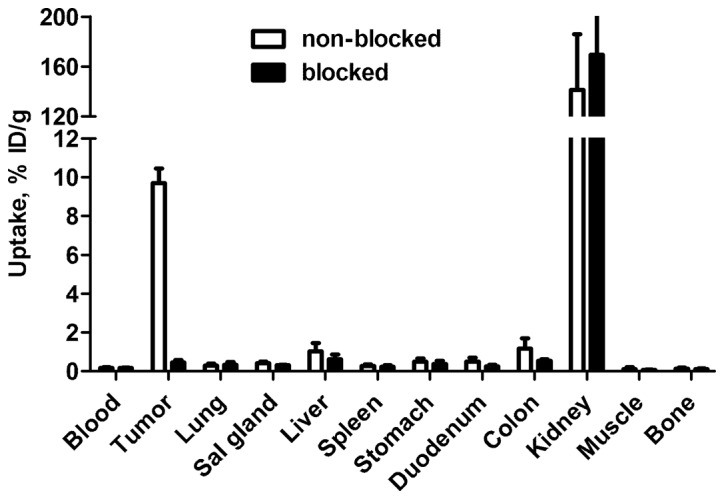
*In vivo* binding specificity of ^99m^Tc-(HE)_3_-ZCAIX:1 in NMRI nu/nu mice bearing SK-RC-52 xenografts at 4 h after injection. Blocked group was subcutaneously preinjected with a large excess amount of unlabeled Affibody. Results are presented as the mean ± standard deviation for 4 animals.

The results of *in vivo* specificity test are presented in Fig. 2. Pre-saturation of CAIX with non-labeled Affibody molecule caused decrease of uptake from 9.7±0.7 to 0.5±0.1% ID/g (P<5×10^−7^). The reduction of tumor uptake demonstrated saturability of the ^99m^Tc-(HE)_3_-ZCAIX:1 tumor accumulation and suggested its specific targeting. There was no significant difference in ^99m^Tc-(HE)_3_-ZCAIX:1 uptake in any other organ after injection of excess amount of non-labeled Affibody molecules.

The data concerning biodistribution of ^99m^Tc-(HE)_3_-ZCAIX:1 (injected dose of 1 μg) in SK-RC-52 xenograft bearing female NMRI nu/nu mice at 1, 4 and 8 h p.i. are shown in Tables III and IV. ^99m^Tc-(HE)_3_-ZCAIX:1 showed a rapid blood clearance already at 1 h p.i. The blood-associated radioactivity reduced ~4 times between 1 and 4 h, but did not change at 8 h after injection in comparison with 4 h. A low level of radioactivity (2.8±1.4% ID at 1 h, 4.5±1.2% ID at 4 h and 1.20±0.30% ID at 8 h p.i.) in the gastrointestinal tract (with its content) suggested that hepatobiliary excretion played a minor role in clearance of ^99m^Tc-(HE)_3_-ZCAIX:1. Most likely, the clearance of the radio-conjugate from body was via glomerular filtration with subsequent re-absorption in kidneys. The tumor uptake of radioactivity was highest at 1 h (22±3% ID/g), which decreased about two times (9.7±0.7% ID/g) at 4 h and remained at the same level at 8 h after injection. There was significant decrease of radioactivity uptake of ^99m^Tc-(HE)_3_-ZCAIX:1 in lung and salivary glands between 1 and 4 h with further decrease between 4 and 8 h after injection. The concentration of radioactivity in all other organs was low at 1 h and decreased significantly by 4 h (P<0.05), but there was no significant difference between 4 and 8 h p.i. On the other hand, there was a significant difference between 1 and 8 h p.i. (P<0.05). Overall, tumor-to-blood and tumor-to-organ (except for tumor-to-colon) ratios of ^99m^Tc-(HE)_3_-ZCAIX:1 were the highest at 4 h (P<0.05), and decreased slightly at 8 h after injection (Table IV).

The data concerning biodistribution study of ^125^I-(HE)_3_-ZCAIX:1 in SK-RC-52 xenograft bearing mice (6 and 8 h p.i.) are presented in Tables V and VI. There was no significant difference between biodistribution and tumor uptake or tumor-to-blood and tumor-to-organ ratio of ^125^I-(HE)_3_-ZCAIX:1 at 6 and 8 h p.i. The tumor and kidney uptake of this radio-conjugate was equal at both 6 h (2.3±0.5% ID/g and 2.7±1.4% ID/g) and 8 h (1.6±0.3% ID/g and 1.6±0.1% ID/g) after injection.

**Figure 3:**
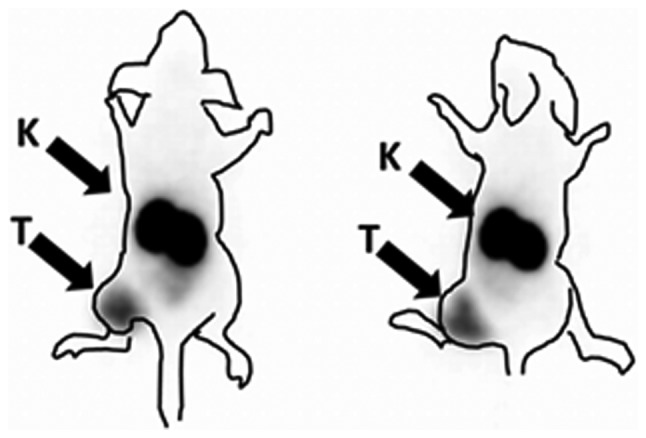
Imaging of CAIX-expressing SK-RC-52 xenografts in NMRI nu/nu mice using clinical gamma-camera. Image was acquired at 4 h after injection of ^99m^Tc-(HE)_3_-ZCAIX:1. Contours were derived from a digital photograph and superimposed over image to facilitate interpretation. Arrows point at tumors (T) and kidneys (K).

The obtained high contrast images of ^99m^Tc-(HE)_3_-ZCAIX:1 confirmed the biodistribution results (Fig. 3). Images obtained at 4 h p.i. showed no visible uptake in any organ except kidneys. Tumors were clearly visualized. The tumor-to-contralateral site ratio was 16.2±0.6.

## Discussion

Imaging contrast is an important factor determining sensitivity and therefore, accuracy of molecular imaging. Data concerning CAIX-targeting antibodies (31) demonstrated that the use of smaller fragments enables better contrast in comparison with intact IgG, and provides shorter time between injection and peak contrast. Affibody molecules are 4-fold smaller than the smallest antibody fragments. An excellent imaging contrast has been demonstrated for a number of molecular targets using Affibody molecules (37). Therefore, we have generated an Affibody molecule with low nanomolar affinity to CAIX. For biodistribution studies, a negatively charged histidine-glutamate-histidine-glutamate-histidine-glutamate [HEHEHE, (HE)_3_]-tag was engineered at N-terminus of the selected Affibody molecules. Previous studies have demonstrated that (HE)_3_-tags enables stable site-specific labeling of Affibody molecules with [^99m^Tc(CO)_3_]^+^, and provide much lower hepatic accumulation of radioactivity than with hexahistidine tags (40). In addition, the use of (HE)_3_-tag provides favorable modification of biodistribution profile even if different labels (e.g. ^111^In or radioidine) are used (41). In the case of anti-CAIX, the use of (HE)_3_-tag enabled efficient and stable labeling using commercially available labeling IsoLink kit. Binding of ^99m^Tc-(HE)_3_-ZCAIX:1 to CAIX-expressing cells was saturable, which indicates its binding specificity. The binding of ^99m^Tc-(HE)_3_-ZCAIX:1 showed a maximum uptake at 2 h after incubation start. An important feature of ^99m^Tc-(HE)_3_-ZCAIX:1 was relatively slow internalization. Only 16.7% of cell-associated radioactivity was internalized at 24 h after incubation start. Interaction of ^99m^Tc-(HE)_3_-ZCAIX:1 with living SK-RC-52 cells showed the presence of two binding sites, one strong (1.3 nM) and another much weaker.

*In vivo*, ^99m^Tc-(HE)_3_-ZCAIX:1 showed efficient and specific targeting of CAIX-expressing SK-RC-52 xenografts, since the tumor uptake was reduced from 9.7±0.7 to 0.4±0.1% ID/g (P<5×10^−7^) by pre-saturation of CAIX *in vivo* (Fig. 2). There was no significant difference in uptake of ^99m^Tc-(HE)_3_-ZCAIX:1 in other organs, suggesting no on-target interaction of ^99m^Tc-(HE)_3_-ZCAIX:1 *in vivo*. This was also confirmed by absence of significant difference in biodistribution after injection of 0.3, 1 or 5 μg ^99m^Tc-(HE)_3_-ZCAIX:1 (Tables I and II). The pattern of ^99m^Tc-(HE)_3_-ZCAIX:1 (Tables III and IV) biodistribution at different time-points was similar to biodistribution of anti-HER2 ^99m^Tc-(HE)_3_-Z_HER2:342_ Affibody molecule (41), which also has no or low target expression in normal tissues. In both cases, there was rapid clearance of radioactivity from blood and normal tissues. At the same time, there was high and non-saturable uptake of radioactivity in kidneys, which is typical for Affibody molecules with residualizing labels. The pattern of tumor uptake *in vivo* resembled binding to the cells *in vitro*: a high uptake at 1 h after injection (23±3% ID/g) was significantly reduced to 9.7±0.7% ID/g at 4 h after injection, but further reduction of tumor uptake to 7.3±3% ID/g at 8 h after injection was not significant. The highest tumor-to-organ ratios for ^99m^Tc-(HE)_3_-ZCAIX:1 were obtained at 4 h after injection. Experimental imaging demonstrated that CAIX-expressing SK-RC-52 xenografts could be visualized with high contrast in mice (Fig. 3). In agreement with biodistribution data, the only organ with higher radioactivity accumulation than in tumor was the kidneys.

One possible clinical application of CAIX imaging is discrimination between benign and malignant primary kidney tumors. This requires higher level of radionuclide accumulation in tumors than in kidneys. Apparently, this is not possible when residualizing labels are used. However, the Affibody molecules showed slow internalization by the tumor cells. Therefore, residualizing properties of a label are not absolutely necessary for a good retention of radionuclides in tumors. On the contrary, internalization of Affibody in kidneys is rapid, and the use of non-residualizing radiohalogen labels results in a rapid washout of radioactivity from kidneys (36,44,41). This created a precondition to obtain a higher radioactivity uptake in tumor than in kidney a few hours after injection for high-affinity anti-HER2 Z_HER2:342_ Affibody molecule or its derivatives (36,44,41). We tested if this is also correct for (HE)_3_-ZCAIX:1 using non-residualizing ^125^I-para-iodobenzoate label. To allow for clearance of radionuclide from kidneys, biodistribution of ^125^I-(HE)_3_-ZCAIX:1 was measured at 6 and 8 h after injection (Table V). As expected, renal accumulation of radioactivity was much lower in the case of radioiodine label than in the case of ^99m^Tc (1.6±0.1 vs. 170±52% ID/g at 8 h p.i., respectively). However, there was also an appreciable release of ^125^I-(HE)_3_-ZCAIX:1 radioactivity from tumors as well. As a result, tumor-to-kidney ratios were 0.90±0.2 at 6 h p.i. and 0.99±0.09 at 8 h p.i., i.e. a positive contrast was not achieved. It has to be noted that anti-HER2 Z_HER2:342_ Affibody molecule has low picomolar affinity (36). It is likely that further affinity maturation of anti-CAIX Affibody molecules might provide variants enabling higher uptake in tumors than in kidneys. With the existing affinities, imaging of primary renal cell carcinoma using Affibody molecules is not feasible. Besides lower renal uptake, the use of radioiodine label did not provide any advantage over ^99m^Tc. The tumor-to-blood ratio was higher for the ^99m^Tc. At 8 h p.i., ^125^I-(HE)_3_-ZCAIX:1 provided higher tumor-to-lung and tumor-to-duodenum ratios than ^99m^Tc-(HE)_3_-ZCAIX:1 at the same time-point. However, these values were not higher than values provided by ^99m^Tc-(HE)_3_-ZCAIX:1 at 4 h after injection.

Earlier, several approaches to develop probes for imaging of CAIX-expression have been evaluated in mice. The use of intact chimeric G250 antibody resulted in a tumor uptake in the range of 20 to 110% ID/g and tumor-to-blood ratio in the range of 4 to 9, depending on xenograft model and labeling chemistry (31,45–46). An optimal imaging time was between 2 and 4 days after injection. The use of Fab and (Fab′)_2_ fragments enabled reduction of imaging time to 24 h p.i., still tumor-to-blood ratio did not exceed 17 (31,32). Attempts to use radiolabeled sulfonamide derivatives did not yet result in development of an imaging agent with tumor uptake of >0.5% ID/g and tumor-to-blood ratios of more than 1 in murine models (47). Current data suggest that the use of ^99m^Tc-(HE)_3_-ZCAIX:1 permits appreciably higher tumor-to-blood ratio than any existing agent for imaging of CAIX *in vivo*. In addition, the optimal imaging time is only a few hours after injection which would facilitate potential clinical use. It opens also an opportunity to use short-lived labels, such as ^68^Ga and ^18^F, for imaging of hypoxia using Affibody molecules in the future.

In conclusion, we show the utility of radiolabeled Affibody molecules as a very promising format for probes for imaging of CAIX-expression *in vivo*. The use of ^99m^Tc-(HE)_3_-ZCAIX:1 permits obtaining the highest tumor-to-blood ratio so far reported in the literature. However, further affinity maturation might be required to provide an Affibody-based agent suitable for imaging of primary renal cell carcinoma.

## Figures and Tables

**Table I:** Biodistribution of ^99m^Tc-(HE)_3_-ZCAIX:1 at 4 h after injection in NMRI nu/nu mice bearing SK-RC-52 xenografts.

	Total injected dose (μg)		
	
Organ	0.3	1	5
Blood	0.2±0.0	0.2±0.0	0.2±0.0
Lung	0.4±0.1	0.3±0.1	0.4±0.1
Salivary gland	0.4±0.1	0.4±0.1	0.5±0.1
Liver	1.2±0.4	1.0±0.4	0.1±0.1
Spleen	0.5±0.1	0.3±0.1	0.4±0.0
Stomach	0.4±0.0	0.5±0.1	0.4±0.1
Duodenum	0.4±0.1	0.5±0.2	0.4±0.1
Colon	0.6±0.3	1.2±0.5	0.9±0.4
Kidney	143±17	141±45	154±2
Tumor	11.0±3.0	10.0±1.0	11.0±2.0
Muscle	0.1±0.0	0.1±0.1	0.1±0.0
Bone	0.2±0.1	0.1±0.0	0.2±0.0

Data are presented as a mean % ID/g value for 4 animals ± standard deviation. There was no significant difference (P>0.05) between ^99m^Tc-(HE)_3_-ZCAIX:1 uptake after injection of 0.3, 1 or 5 μg.

**Table II:** Tumor-to-organ ratios of ^99m^Tc-(HE)_3_-ZCAIX:1 at 4 h after injection in NMRI nu/nu mice bearing SK-RC-52 xenografts.

	Dose (μg)		
	
Organ	0.3	1	5
Blood	62±14	53±10	65±5
Lung	26±6	35±11	24±7
Salivary gland	27±5	25±6	23±2
Liver	9±2	11±4	11±1
Spleen	23±1^a^	36±14	29±4
Stomach	29±5	20±6	29±4
Duodenum	30±5	22±8	26±1
Colon	19±8	9±3	14±5
Kidney	0.1±0.0	0.1±0.0	0.1±0.0
Muscle	95±15	104±52	102±30
Bone	47±9	80±32	59±23

Data are presented as a mean value for 4 animals ± standard deviation.

a Significant difference (P<0.05) between tumor-to-organ ratios after injection of 0.3 and 5 μg.

**Table III:** Biodistribution of ^99m^Tc-(HE)_3_-ZCAIX:1 (injected dose 1 μg) at 1, 4 and 8 h after injection in NMRI nu/nu mice bearing SK-RC-52 xenografts.

	^99m^Tc-(HE)_3_-ZCAIX:1		
	
	1 h	4 h	8 h
Blood	0.9±0.2^a^	0.2±0.0	0.2±0.0^c^
Lung	1.4±0.3^a^	0.3±0.1^b^	0.6±0.2^c^
Salivary gland	1.0±0.2^a^	0.4±0.1^b^	0.85±0.2
Liver	4.0±0.7^a^	1±0.4	0.9±0.3^c^
Spleen	0.7±0.1^a^	0.3±0.1	0.6±0.2
Stomach	1.4±0.4^a^	0.5±0.1	0.5±0.3
Duodenum	2.6±0.5^a^	0.5±0.2	0.5±0.2^c^
Colon	0.7±0.1	1.2±0.5	0.5±0.1
Kidney	226±20^a^	141±45	170±52
Tumor	22.3±3.2^a^	9.7±0.7	7.3±3.0^c^
Muscle	0.4±0.1^a^	0.1±0.1	0.2±0.1
Bone	0.5±0.1^a^	0.1±0.0	0.3±0.2

Data are presented as a mean % ID/g value for 4 animals ± standard deviation. (Data for intestines with content is presented as % ID/sample).

a Significant difference (P<0.05) between uptake of ^99m^Tc-(HE)_3_-ZCAIX:1 at 1 and 4 h after injection.

b Significant difference (P<0.05) between uptake of ^99m^Tc-(HE)_3_-ZCAIX:1 at 4 and 8 h after injection.

c Significant difference (P<0.05) between uptake of ^99m^Tc-(HE)_3_-ZCAIX:1 at 1 and 8 h after injection.

**Table IV:** Tumor-to-organ ratios of ^99m^Tc-(HE)_3_-ZCAIX:1 (injected dose 1 μg) at 1, 4 and 8 h after injection in NMRI nu/nu mice bearing SK-RC-52 xenografts.

	^99m^Tc-(HE)_3_-ZCAIX:1		
	
	1 h	4 h	8 h
Blood	26±4^a^	53±1	42±7^c^
Lung	15±3^a^	35±11^b^	13±1^c^
Salivary gland	23±6	25±6^b^	10±5^c^
Liver	6±1^a^	11±4	9±0.3^c^
Spleen	29±1	36±14^b^	14±4^c^
Stomach	17±5	20±6	19±10^c^
Duodenum	9±3^a^	22±8	15±3^c^
Colon	32±5^a^	9±3	14±3^c^
Kidney	0.1±0.0	0.1±0.0	0.0±0.0^c^
Muscle	61±14	104±52	45±8^c^
Bone	47±7	80±32^b^	30±10^c^

Data are presented as a mean value for 4 animals ± standard deviation.

a significant difference (p<0.05) between uptake of ^99m^Tc-(HE)_3_-ZCAIX:1 at 1 and 4 h after injection.

b significant difference (p<0.05) between uptake of ^99m^Tc-(HE)_3_-ZCAIX:1 at 4 and 8 h after injection.

c significant difference (p<0.05) between uptake of ^99m^Tc-(HE)_3_-ZCAIX:1 at 1 and 48 h after injection.

**Table V:** Biodistribution of ^125^I-(HE)_3_-ZCAIX:1 (injected dose 1 μg) at 6 and 8 h after injection in NMRI nu/nu mice bearing SK-RC-52 xenografts.

	^125^I-(HE)_3_-ZCAIX:1	
	
	6 h	8 h
Blood	0.09±0.02	0.07±0.02
Lung	0.09±0.04	0.05±0.01
Salivary gland	0.10±0.04	0.11±0.05
Liver	0.20±0.01	0.18±0.01
Spleen	0.08±0.02	0.08±0.01
Stomach	0.08±0.03	0.08±0.06
Duodenum	0.07±0.01	0.05±0.03
Colon	0.04±0.01	0.03±0.01
Kidney	2.7±1.4	1.6±0.1
Tumor	2.2±0.5	1.6±0.3
Muscle	0.03±0.01	0.022±0.04
Bone	0.04±0.01	0.04±0.03

Data are presented as a mean % ID/g value for 3 animals ± standard deviation.

**Table VI:** Tumor-to-organ ratios of ^125^I-(HE)_3_-ZCAIX:1 (injected dose 1 μg) at 6 and 8 h after injection in NMRI nu/nu mice bearing SK-RC-52 xenografts.

	^125^I-(HE)_3_-ZCAIX:1	
	
	6 h	8 h
Blood	26±2	24±5
Lung	30±13	31±5
Salivary gland	22±1	18±11
Liver	11±2	9±1
Spleen	31±12	20±6
Stomach	30±11	28±14
Duodenum	33±6	35±16
Kidney	0.9±0.2	1.0±0.1
Muscle	84±18	77±18
Bone	53±11	56±37

Data are presented as a mean % ID/g value for 3 animals ± standard deviation.
